# Targeting and Sensitization of Breast Cancer Cells to Killing with a Novel Interleukin-13 Receptor α2-Specific Hybrid Cytolytic Peptide

**DOI:** 10.3390/cancers15102772

**Published:** 2023-05-16

**Authors:** Riaz Jannoo, William Walker, Venkateswarlu Kanamarlapudi

**Affiliations:** 1 UCL ECMC GCLP Facility, UCL Cancer Institute, University College London, London WC1E 6DD, UK; m.jannoo@ucl.ac.uk; 2 Institute of Life Science, School of Medicine, Swansea University, Singleton Park, Swansea SA2 8PP, UK; w.walker@swansea.ac.uk

**Keywords:** IL-13Rα2, TNBC, cytolytic peptide, cytotoxic, adjuvant, epigenetic, cancer therapy

## Abstract

**Simple Summary:**

Breast cancer is the most commonly diagnosed cancer and the second leading cause of cancer deaths among women globally. Due to its biological heterogeneity, breast cancer treatment and prognosis are highly variable among patients. In particular, the triple-negative subtype (TNBC) has a poorer prognosis with a lack of targeted therapies. With this in mind, we sought to develop a potentially more effective, novel receptor-targeting strategy. This study describes measuring the expression of the putative biomarker, interleukin-13 receptor (IL-13R)α2, in breast cancer (including TNBC) and its therapeutic targeting using a novel hybrid cytolytic peptide (Pep-1-Phor21) approach. We have shown in this manuscript that IL-13Rα2 exhibits potential as a therapeutic target, particularly for TNBC types of breast cancer. Importantly, drug-induced breast cancer cell lysis could be enhanced by treatment with epigenetically active anti-cancer compounds, suggesting that a combination adjuvant therapy of Pep-1-Phor21 with such compounds may be a particularly productive strategy for TNBC.

**Abstract:**

Highly metastatic breast cancers, such as triple-negative subtypes (TNBC), require the most effective treatments. Since interleukin-13 receptor (IL-13R)α2 is reportedly over-expressed in some cancers, we investigated here its expression and the feasibility of therapeutically targeting this receptor in breast cancer using a novel hybrid cytolytic peptide (Pep-1-Phor21) consisting of IL-13Rα2-binding (Pep-1) and cytolytic (Phor21) domains. This study demonstrates that particularly TNBC tissues and cells display the prominent expression of IL-13Rα2. Furthermore, Pep-1-Phor21 induced the rapid necrosis of tumor cells expressing cell-surface IL-13Rα2. Notably, IL-13Rα2 expression was found to be epigenetically regulated in breast cancer cells in that the inhibition of histone deacetylase (HDAC) or DNA methyltransferase (DNMT) upregulated IL-13Rα2 expression, thereby sensitizing them to Pep-1-Phor21. IL-13Rα2-negative non-malignant cells were refractory to these epigenetic effects. Consistent with its cytolytic activity, Pep-1-Phor21 readily destroyed IL-13Rα2-expressing breast cancer spheroids with HDAC or DNMT inhibition, further enhancing cytolytic activity. Therefore, the Pep-1-Phor21-mediated targeting of IL-13Rα2 is a potentially novel therapeutic strategy for TNBC. Given that tumor cells can be selectively sensitized to Pep-1-Phor21 via the epigenetic up-regulation of IL-13Rα2, a combined adjuvant approach involving Pep-1-Phor21 and epigenetic inhibitors may be an effective strategy.

## 1. Introduction

Breast cancer accounts for one of the commonest forms of malignancy in women and while advances in treatments have decreased mortality rates for many forms of breast cancer, highly metastatic types such as triple-negative breast cancer (TNBC) remain a considerable therapeutic challenge [1]. In particular, as TNBC-type tumors lack the expression of the estrogen receptor (ER), the progesterone receptor (PR), and human epidermal growth factor (EGF) receptor-2 (HER2), current treatments, therefore, consist of adjuvant-type approaches based on agents that include anthracyclines, taxanes, alkylating agents, and anti-metabolites [2,3]. However, these approaches lack tumor-specific targeting and produce considerable side effects and, consequently, TNBC is associated with faster relapse times and higher mortality rates [4,5]. Moreover, the development of multi-drug resistance, where (for example) cancer cells over-express intracellular drug-efflux pumps, further reduces the effectiveness of conventional cytotoxic chemotherapies [6]. Therefore, more selective and effective targeted therapies for TNBC-type breast cancer are urgently required.

More selective approaches to tumor therapy include targeting receptors that are upregulated in an oncogenic setting with one putative candidate being the cell-surface protein, interleukin-13 receptor α2 (IL-13Rα2). Previous studies have shown that IL-13Rα2 is over-expressed in the latter stages of breast cancer, where increased expression levels correlate with higher rates of metastasis and a poorer prognosis [7,8]. Further, IL-13Rα2, which is also called cancer/testis antigen (CT)19, has insignificant expression in normal tissues [9,10]. Initially, IL-13Rα2 was characterized as a decoy receptor, where it binds with high affinity to its natural cytokine ligand, IL-13 [11,12]. However, recent studies have shown a more complex signaling function for IL-13Rα2 in brain tumor progression [12]. Although the functional significance of IL-13Rα2 expression on tumor cells remains largely undefined, under certain circumstances, IL-13Rα2 signaling can mediate a variety of cellular and tissue responses that may impact tumorigenesis [13]. Nevertheless, various IL-13Rα2-targeting strategies involving cytotoxic therapeutics have been developed [14]. For example, IL-13 conjugated with *Pseudomonas* exotoxin subunits (IL-13-PE) has been developed for glioblastoma therapy [15]. However, treatment-induced neurotoxicity associated with the internalization of IL-13-PE [16,17] produced off-target tissue damage, attributed to the ability of IL-13-PE to bind to the alternative physiological receptor for IL-13, namely the IL-13Rα1/IL-4Rα heterodimer [18]. Therefore, in pursuing a strategy of targeting IL-13Rα2-expressing tumors, candidate drugs must display appropriate specificity and selectivity. Furthermore, IL-13Rα2 expression is regulated via epigenetic mechanisms [19,20] and, concerning breast cancer, epigenetic activity, including DNA methylation and histone modifications, has been linked to disease progression [21,22,23]. Notably, ER expression can be re-established in breast cancer cells after treatment with histone deacetylase (HDAC) inhibitors [23], raising the possibility that similar mechanisms may also upregulate IL-13Rα2 expression, consequently sensitizing them to IL-13Rα2-targeting strategies.

To improve selectivity and specificity in targeting IL-13Rα2, we developed a hybrid cytolytic peptide (Pep-1-Phor21), which, unlike previous strategies, is not a cytotoxin–drug conjugate. Instead, Pep-1-Phor21 consists of a unitary peptide structure bearing distinct receptor-binding (Pep-1) and cytolytic (Phor21) domains. The seven-amino-acid Pep-1 peptide was originally discovered by screening a phage display library for novel IL-13Rα2 ligands and it selectively binds IL-13Rα2 with high affinity at a site on the receptor distinct from that utilized for IL-13 binding [24]. Pep-1 in conjugate form has been used as drug therapy and as a means of PET imaging IL-13Rα2-expressing gliomas [25,26]. The Phor21 lytic domain corresponds to the previously described amphipathic membrane-disrupting cytolytic peptide, which contains three sequence repeats of seven amino acids (KFAKFAK) [27]. This cytolytic peptide kills cells through necrosis by acting on the plasma membrane and, therefore, bypasses multidrug resistance [28]. Further, unlike the lytic peptides that kill cells by apoptosis, the Phor21 cytolytic peptide doesn’t internalize since it elicits necrosis [29]. Although the combination of Pep-1 with Phor21 (Pep-1-Phor21) has not previously been investigated, Phor21, when combined with a segment of the β-chain of chorionic gonadotropin (β-CG) (Phor21-βCG[ala]), was reportedly able to target cancer cells expressing the luteinizing hormone/chorionic gonadotropin receptor (LHCGR) [30]. Since Phor21-βCG[ala] has been shown to have a short in vivo half-life (~5 h), it is very unlikely to elicit an immune response and/or cause liver toxicity [28]. We have demonstrated recently that the Pep-1-Phor21 cytolytic peptide shows anticancer properties against IL-13Rα2-expressing prostate cancer cells [31].

This study addresses the hypothesis that IL-13Rα2 is a druggable target for the treatment of breast cancer. To test this, we examined IL-13Rα2 expression in both non-TNBC and TNBC breast cancer using representative cell lines, and tissue cDNA- and micro-arrays. Further, we defined the specific activity of Pep-1-Phor21 against IL-13Rα2-expressing breast cancer cells and -transfected cells. To model more tumor-representative conditions, we determined in detail the activity of Pep-1-Phor21 against IL-13Rα2-positive TNBC spheroid cultures [32]. To sensitize breast cancer cells to subsequent Pep-1-Phor1 treatment, we also examined the epigenetic regulation of IL-13Rα2 expression in breast cancer cells with a specific focus on HDAC and DNMT inhibitor treatments. Taken together, the results show that TNBC cells, in particular, express relatively high levels of IL-13Rα2 and the Pep-1-Phor21 peptide efficiently targets these tumor cells, rapidly destroying them through cytolysis. Furthermore, HDAC or DNMT inhibition selectively upregulated IL-13Rα2 expression and enhanced the effectiveness of Pep-1-Phor21 against tumor cells, raising the possibility that a combination drug therapy approach may be particularly effective.

## 2. Materials and Methods

### 2.1. Cell Culture

Human non-tumorigenic breast epithelial cell line (MCF-10A (catalog number (#) CRL-10317)), breast cancer cell lines (MCF-7 (#HTB-22), MDA-MB-231 (#HTB-26)) and COS-7 cell line (#CRL-1651) were obtained from the American Type Culture Collection (ATCC^®^). LM2, the highly metastatic 1834 sub-line of MDA-MB-231, was kindly donated by Dr. J. Massague, Memorial Sloan-Kettering Cancer Center, NY [7]. COS-7, MCF-7, and MDA-MB-231/LM2 cell lines were maintained in Dulbecco’s Modified Eagles Medium (DMEM) supplemented with 10% fetal bovine serum (FBS) and 1% PSG (200 mM L-glutamine, 10,000U/mL penicillin and 10mg/mL streptomycin). MCF-10A cells were maintained in Ham’s F12:DMEM (50:50) culture medium containing 5% horse serum, 1% PSG, 20 ng/mL EGF, 0.1 µg/mL cholera toxin, 10 µg/mL insulin, and 0.5 µg/mL hydrocortisone [33].

To create spheroids, a Terasaki plate (Greiner Bio-One (#653180), Stonehouse, UK) seeded with cells (20 µL of 1 × 10^5^ cells/mL per well) was incubated upside-down in a humidified incubator. Where appropriate, cells were labeled with Vybrant^®^ DiO fluorescent vital membrane dye (Thermo-Fisher Scientific (#V22886), Loughborough, UK). After 1 day of incubation, the spheroids, along with the medium pooled from 3 wells of the Terasaki plate, were transferred into a single well of a U-bottom, surface-repellent, 96-well plate (Greiner Bio-One (#650970)). Spheroid formation was monitored using an inverted light microscope and viability was assessed using LIVE/DEAD^®^ Viability/Cytotoxicity Assay kit (Thermo-Fisher Scientific (#L3224)), which contains ethidium homodimer-I (EthD-1). Labeled spheroids were pipetted into pre-prepared 50% (*v*/*v*) Matrigel^®^ (Merck (#E1270), Gillingham, UK): 30 µL Matrigel^®^ per well/96-well μClear^®^ half area black plate with a flat bottom (Greiner Bio-One (#675090)), pre-incubated for 24 h to allow the solidification of the Matrigel^®^. Spheroids in Matrigel^®^ were then treated with a test compound for up to 3 h and then incubated in phenol red-free RPMI-1640 (Thermo-Fisher Scientific (#11835030)) containing 2 µM EthD-1 at room temperature (RT) for 40 min. Spheroids in Matrigel^®^ were then washed three times with phenol red-free RPMI-1640 and immediately imaged by confocal microscopy (LSM 710, Carl Zeiss, Inc., White Plains, NY, USA). Where applicable, COS-7 cells were transfected for 48 h with expression plasmid IL-13Rα2-mCherry [11] or Myc-LHCGR [34] or an empty control plasmid (mCherry) using jetPRIME^®^ transfection reagent (Polyplus-transfection SA (#101000015), Illkirch-Graffenstaden, France) according to the manufacturer’s instructions [35].

### 2.2. Peptides and Other Reagents

Pep-1-Phor21 (ACGEMGWVRCGGGKFAKFAKKFAKFAKKFAKFAK) and its individual subunit motifs, Pep-1 (ACGEMGWVRCGGGS) and Phor21 (KFAKFAKKFAKFAKKFAKFAK), were synthesized (>95% purity) by Thermo Fisher Scientific Inc. Phor21-βCG [30] was used where indicated. Epigenetically active inhibitor compounds Trichostatin-A (TSA (#T8552)) and 5-aza-2′-deoxycytidine (5-aza-dC (#A3656)) were obtained from Merck.

### 2.3. Real-Time-PCR (RT-PCR)

Total RNA was isolated from cultured cells using RNeasy mini kits (Qiagen (#74104), Manchester, UK). An amount of 1 µg of total RNA was reverse transcribed to cDNA in a 20 µL reaction volume using a High-Capacity cDNA Reverse Transcription Kit (Applied Biosystems (#4368814)). RT-PCR was carried out using the SensiFAST SYBR^®^ and Fluorescein Kit (Bioline (#BIO-96005)) in a final volume of 10 µL containing 0.5 µL cDNA and 250 nM primers and a Bio-Rad CFX 96 Real-Time Detection System. Primer sequences were 5′-TAACCTGGTCAGAAGTGTGCC-3′ (sense) and 5′-GGAGGGTTAACTTTTATACTCGGTGT-3′ (antisense) for IL-13Rα2 and 5′-CAGCCATGTACGTTGCTATCCAGG-3′ (sense) and 5′-AGGTCCAGACGCAGGATGGCATG-3′ (antisense) for beta-actin. The relative expression of IL-13Rα2 mRNA was calculated using the 2^−ΔΔCt^ method with beta-actin mRNA as the internal control [36]. To determine relative IL-13Rα2 mRNA expression in breast cancer tumors representing various stages of the disease, a similar RT-PCR methodology was applied to Origene TissueScan™ Breast Cancer cDNA Arrays (samples from CSRT104, BCRT103, and BCRT104; 60 tumor samples (*n* = 60) covering various disease stages with non-malignant (*n* = 7) breast tissue cDNA samples for comparison) [37].

### 2.4. Immunohistochemistry

For the detection of IL-13Rα2 expression in primary tumor tissue, immunohistochemical staining was performed on a breast cancer tissue array (BR1009, US Biomax) using a Vectastain^®^ Elite ABC-HRP kit (Vector Laboratories (#PK-6200)) [38]. Briefly, duplicate cores on the array (formaldehyde-fixed tissue, paraffin sections) were first pre-treated with 1.0% hydrogen peroxide to block endogenous peroxidase activity followed by antigen un-masking with Retrievagen A solution (BD Biosciences (#BD 550524)). Subsequent sequential blocking treatments included incubation with 1.5% normal horse serum and avidin/biotin solution (Vector Laboratories (#SP-2001)). IL-13Rα2 was detected using mouse anti-human IL-13Rα2 (2K8, sc-134363, Santa Cruz Biotechnology Inc., diluted 1:200 (0.5 µg/mL final concentration)) primary antibody, biotinylated secondary antibody (1:500 dilution), and horseradish peroxidase (HRP)-conjugated avidin (1:500 dilution). Diaminobenzidine (DAB) enzyme substrate was used to visualize anti-IL-13Rα2 staining. The manual scoring of tissue cores for positive staining was based on the Allred scoring system in which the percentage of positive cells (proportion score: 0 [none]–5 [100%]) and the intensity of the reaction product (intensity score: 0 [none]–3 [strong]) in the entire tissue core were evaluated [36]. The two scores were added together for a final score with a maximum score (strongly positive) of 8 and negative staining = 0.

### 2.5. Immunoblotting

Harvested cells were lysed with standard RIPA lysis buffer (10 mM Tris-HCl pH 7.5, 10 mM EDTA, 1% NP-40, 0.1% SDS, 150 mM NaCl, 0.5% sodium deoxycholate) containing 1% mammalian proteinase inhibitor mix. Cell lysates fractionated by SDS-PAGE gel electrophoresis were transferred onto polyvinylidene fluoride (PVDF) membranes as described [39]. IL-13Rα2 protein was detected with an anti-IL-13Rα2 mouse monoclonal antibody (sc-134363, Santa Cruz Biotechnology) diluted 1:500 in blocking buffer (TBS, 0.1% Tween-20 containing 5% milk powder) followed by incubation with an HRP-conjugated anti-mouse secondary antibody. Where indicated, mCherry was detected with rabbit anti-RFP antibody (Abcam [#ab34771]). Membranes were developed using ECL Select Substrate (GE Healthcare (#RPN2235), Hatfield, UK) and bands were visualized using a Bio-Rad ChemiDoc TM XRS system (Watford, UK). For standardization, blots were stripped as described [40] and re-probed using an anti-α tubulin mouse monoclonal antibody (Merck (#T6074), Gillingham, UK).

### 2.6. Cell Surface Enzyme-linked Immunosorbent Assay (ELISA)

The cell surface expression of IL-13Rα2 was assessed by ELISA using non-permeabilized cells, as previously described [34,41]. Briefly, cells were plated at 60–80% confluence in poly-L-lysine-coated wells of a 48-well plate. After 24 h in culture, cells were serum-starved for 2 h and then fixed with 4% (*w*/*v*) paraformaldehyde for 5 min. Following incubation with blocking buffer (1% bovine serum albumin (BSA) in TBS), cells were incubated with an anti-IL-13Rα2 mouse monoclonal antibody (sc-134363, Santa Cruz Biotechnology; diluted 1:800) followed by incubation with an HRP-conjugated anti-mouse IgG antibody. Following washing, surface staining was developed by incubating with 1-step Ultra TMB-ELISA substrate solution (Thermo-Fisher Scientific (#34029)) and absorbance was determined at 450 nm.

### 2.7. Cell Viability, Cytotoxicity, and Apoptosis Assays

Unless specified otherwise, 96-well black, µClear, half area, and flat-bottom plates (Greiner Bio-One (#675090)) were used for these assays. Cell viability was assessed using Alamar Blue according to the manufacturer’s instructions (Thermo Fisher Scientific (#DAL1100)). Before this, the optimal cell density of MCF-10A, MCF7, and MDA-MB-231 required for cell viability was established as 40,000 cells per well (Figure S2). This was carried out by plating 10,000 to 80,000 cells per well and analyzing cell growth daily for up to 4 days by using Alamar Blue assay. Cell cytotoxicity was assessed using CellTox™ Green Cytotoxicity Assay (Promega (#G8743), Southampton, UK). Briefly, 40,000 cells per well were plated. After 24 h, the medium was replaced with medium containing 0.1% (*v*/*v*) CellTox Green Dye and the appropriate test compound. Fluorescence was measured using the 490 nm (excitation) and 525 nm (emission) settings on a POLARstar Omega microplate reader (BMG LABTECH). Fluorescence after the initial 30 min was considered zero, with subsequent fluorescence measurements obtained at 3 h intervals. Cells treated with the lysis buffer provided in the assay kit were used as the positive control (100% cytotoxicity). The combined measurement of cell viability, cytotoxicity, and apoptosis was determined by ApoTox-Glo^TM^ Triplex Assay (Promega (#G6320)): Briefly, 40,000 cells per well were plated into the 96-well µClear^®^ half area black (for the cell viability and cytotoxic assays) or white (for the apoptosis assay) plate with a flat bottom (Greiner Bio-One (#655083)). After 24 h, the medium was replaced with fresh medium containing the test compound. After 6 h, 10 µL of cell viability/cytotoxicity reagent was added to each well and incubated for a further 1 h at 37 °C. Cell viability (fluorescence) was measured at 400 nm (excitation) and 505 nm (emission) settings. Cytotoxicity was measured at 485 nm (excitation) and 520 nm (emission). Apoptosis (luminescence) was determined by adding Caspase-Glo^®^ 3/7 reagent and incubating for 30 min at RT.

### 2.8. Statistical Analysis

Data were analyzed using GraphPad Prism version 6.0 software. All data are presented as the mean ± standard error of the mean (SEM) from three independent experiments [42]. Statistical analysis was conducted using an unpaired Student’s *t*-test or one-way analysis of variance with post-Tukey’s multiple comparisons test with a significance of * *p* ≤ 0.05, ** *p* ≤ 0.01, *** *p* ≤ 0.001, **** *p* ≤ 0.0001; ns = not significant.

## 3. Results

### 3.1. Expression of IL-13Rα2 in Breast Cancer

**Figure 1:**
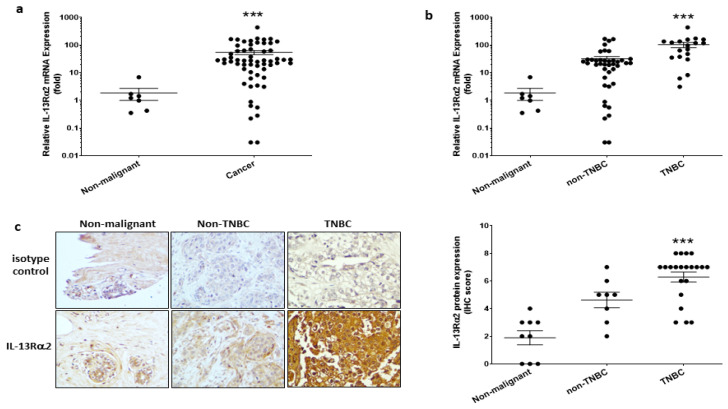
Expression of IL-13Rα2 in breast cancer tissue. (**a**) RT-PCR analysis of IL-13Rα2 mRNA expression in cDNA array samples (TissueScan™, Origene) derived from breast cancer (number of samples (*n* = 60)) and non-malignant (*n* = 7) tissue. Shown is the fold-change relative to mean expression in non-malignant samples (mean fold-change, cancer versus non-malignant; *** *p* ≤ 0.001). (**b**) Stratification analysis of IL-13Rα2 mRNA expression data comparing TNBC-type (*n* = 19) versus non-TNBC (*n* = 41) tumors (mean fold-change, TNBC versus non-TNBC, *** *p* ≤ 0.001). (**c**) Immunohistochemical analysis of IL-13Rα2 protein expression in breast cancer tissue array samples (US Biomax, BR1009). Shown images (at 40× magnification) are representative examples of specific staining with an anti-IL-13Rα2 antibody (sc-134363) versus a mouse IgG2a isotype control: (**left panel**), non-malignant tissue; (**middle panel**) non-TNBC tumor (ER+, stage IIa); (**right panel**) TNBC tumor (stage IIb). (**Right Figure**), scoring analysis of anti-IL-13Rα2 reactivity in non-malignant (*n* = 9), non-TNBC (*n* = 8), and TNBC (*n* = 21) tissue array sections (mean score, TNBC versus non-malignant, *** *p* ≤ 0.001).

For the determination of IL-13Rα2 mRNA expression, real-time RT-PCR was performed on breast cancer cDNA arrays containing multiple samples representative of the diverse breast cancer phenotypes. IL-13Rα2 mRNA expression was found to be significantly higher in breast cancer tissues compared with that in non-malignant breast tissues (Figure 1a). Further, IL-13Rα2 mRNA expression was significantly greater in TNBC-type tumors, compared to that in non-TNBC tumors (positive for at least one of ER, PR, HER2) or non-malignant breast tissue (Figure 1b). Immunohistochemical staining of tissue micro-array samples confirmed this pattern of expression at the protein level in that IL-13Rα2 expression was more predominantly detectable in TNBC-type tumors (Figure 1c).

**Figure 2:**
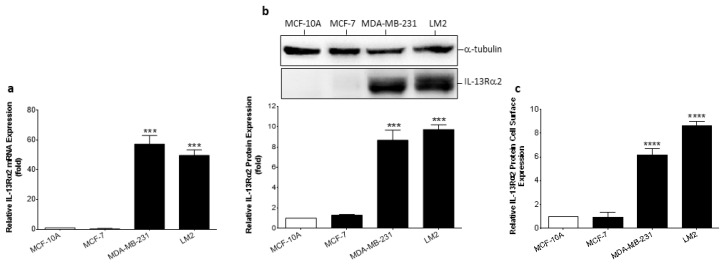
Expression of IL-13Rα2 in breast cancer cell lines. (**a**) RT-PCR analysis of IL-13Rα2 mRNA expression in non-malignant (MCF-10A), non-TNBC (MCF-7), and TNBC (MDA-MB-231, LM2) cells. (**b**) Quantification of IL-13Rα2 protein expression in representative cell lines by Western blot. For densitometry, individual band density was normalized against α-tubulin expression. The uncropped bolts are shown in Supplementary Materials File S1. (**c**) Cell-surface expression of IL-13Rα2 protein was measured in non-permeabilized cells by cell-based ELISA. All graphs show fold-change relative to mean expression in MCF-10A cells. Data = mean values ± SEM of three independent experiments (*** *p* ≤ 0.001; **** *p* ≤ 0.0001).

To validate cells for in vitro modeling studies, IL-13Rα2 expression was determined in the non-tumorigenic breast epithelial cell line (MCF-10A), a non-TNBC cell line (MCF-7), and two TNBC cell lines (MDA-MB-231 and its highly-metastatic sub-line, LM2 [7]). RT-PCR analysis revealed that only the TNBC-type cell lines had significantly elevated expression of IL-13Rα2 mRNA (Figure 2a) and this was confirmed at the protein level by immunoblotting (Figure 2b). In contrast, non-malignant MCF-10A and non-TNBC MCF-7 cancer cells did not exhibit detectable IL-13Rα2 protein expression. A cell-based ELISA was utilized to determine whether the IL-13Rα2 protein was appropriately localized at the cell surface and this analysis confirmed that IL-13Rα2 cell-surface expression was undetectable in MFC-10A and MCF-7 cells (Figure 2c). In comparison, TNBC-type cell lines exhibited prominent cell-surface IL-13Rα2 expression (MDA-MB-231: 613.3% ± 55.3, LM2: 863.5% ± 33.0; relative to MCF-10A). Therefore, IL-13Rα2 expression is similar in breast cancer tissue samples and representative cell lines with an expression more prominent in TNBC-type tumor cells.

### 3.2. Evaluation of Pep-1-Phor21-Mediated Cell Targeting

**Figure 3:**
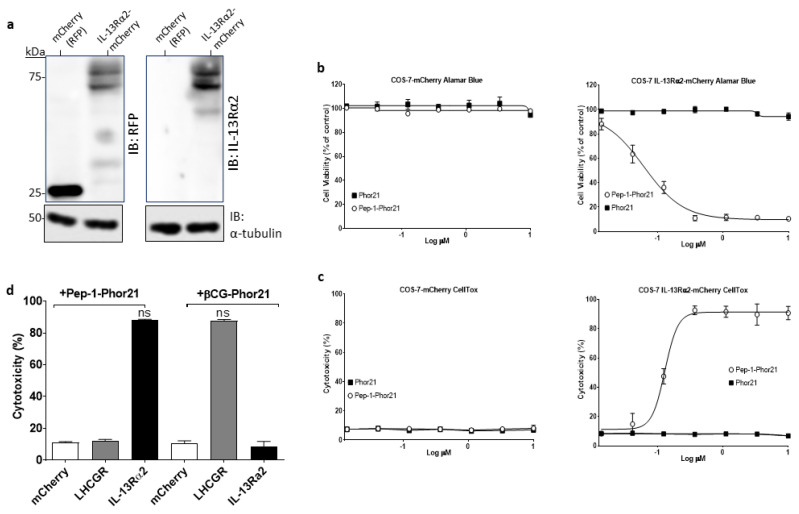
Evaluation of Pep-1-Phor21 activity. (**a**) Protein samples from transfected COS-7 cells expressing the red fluorescent protein (RFP) mCherry (27kDa) or IL-13Rα2-mCherry (75 kDa) were immunoblotted (IB) using either an anti-RFP or an anti-IL-13Rα2 antibody with α-tubulin detection as a loading control. The uncropped bolts are shown in Supplementary Materials File S1. (**b**) COS-7 cells transfected with IL-13Rα2-mCherry plasmid or empty mCherry vector were treated with Pep-1-Phor21 (O) or Phor21 (∎) at 0–10 µM for 3 h and their viability was determined by Alamar Blue assay (IC_50_ for Pep-1-Phor21 against IL-13Rα2-transfected cells = 0.133 µM ± 0.04). (**c**) CellTox assay was used to determine the cytotoxic activity of Pep-1-Phor21 against IL-13Rα2-transfected cells (IC_50_ = 0.130 µM ± 0.03). (**d**) IL-13Rα2- or LHCGR-transfected COS-7 cells were treated with 0.5 µM Pep-1-Phor21 or βCG-Phor21 for 3 h and their relative cytotoxicity was determined (versus empty vector-transfected cells). Only IL-13Rα2-transfected cells exhibited susceptibility to the cytotoxic effects of Pep-1-Phor21, whilst remaining resistant to βCG-Phor21. Data = mean value ± SEM of three independent experiments (ns = not significant).

To evaluate the specificity and activity of Pep-1-Phor21, COS-7 cells transfected with either an empty mCherry vector or a mCherry-tagged IL-13Rα2 expression plasmid were used. Immunoblotting analysis confirmed that appropriately transfected cells expressed either the ~27 kDa mCherry protein or the ~75 kDa IL-13Rα2 mCherry-fusion protein (Figure 3a). Transfected cells were subsequently treated with Pep-1-Phor21 (0–10 µM) for 3h and evaluated cell viability (Alamar Blue assay; Figure 3b) and cytotoxicity (CellTox assay; Figure 3c). Pep-1-Phor21 was shown to kill IL-13Rα2-expressing COS-7 cells in a dose-dependent manner with a determined 50% inhibitory concentration (IC_50_) of 0.13µM for both Alamar Blue and CellTox assays. A peptide consisting of only the subunit lytic motif (Phor21) exhibited no appreciable activity against IL-13Rα2-expressing or mCherry-expressing COS-7 cells. To further define specificity, we compared Pep-1hor21 with an LHCGR-targeting hybrid peptide (Phor21-βCG) [30] in their ability to specifically kill IL-13Rα2- or LHCGR-transfected COS-7 cells (Figure 3d). Pep-1-Phor21 had no demonstrable cytotoxic effect on COS-7 cells expressing LHCGR (COS-7 LHCGR), whereas only Phor21-βCG exhibited significant cytotoxic activity against COS-7 LHCGR cells (cytotoxicity = 86.2% ± 0.6 of maximal activity). Reciprocally, only COS-7 cells expressing IL-13Rα2 (COS-7 IL-13Rα2) were susceptible to Pep-1-Phor21 treatment (cytotoxicity = 83.3% ± 3.4). Therefore, Pep-1-Phor21 does not exhibit any appreciable off-target activity, and the complete unitary peptide structure is required for functional activity against IL-13Rα2-expressing cells.

### 3.3. Cytotoxic Activity of Pep-1-Phor21 against IL-13Rα2-Positive Breast Cancer Cells

**Figure 4:**
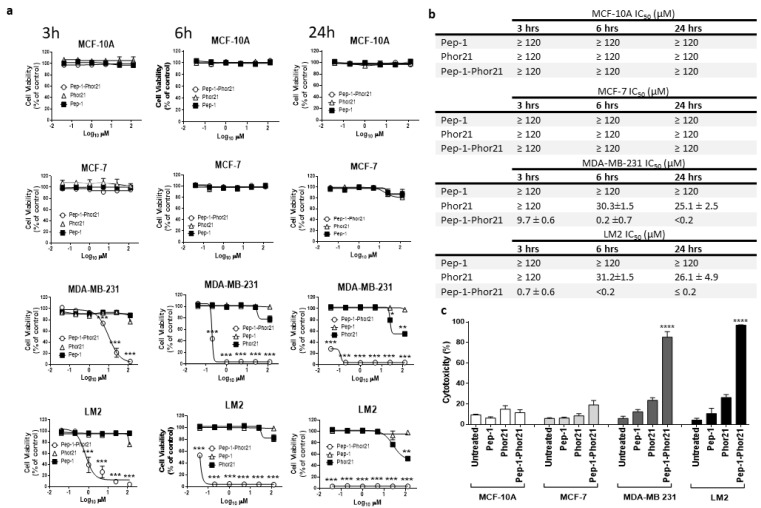
Treatment of representative breast cancer cell lines with Pep-1-Phor21. (**a**) Dose-dependent effect of Pep-1-Phor21 (O), Pep-1 (■), or Phor21 (∆) on the viability of non-tumorigenic (MCF-10A), non-TNBC (MCF-7), and TNBC cells (MDA-MB 231, LM2). Cells were treated with different concentrations of Pep-1-Phor21 (effective concentration range = 0–120 µM, a 5-fold serial dilution) and their viability was assessed after 3 h, 6 h, or 24 h of the treatment by Alamar Blue assay. (**b**) The IC_50_ of peptides for various cell lines for different incubation times. (**c**) The cytotoxic effect of individual peptides on cell lines was also assessed by CellTox assay. MCF-10A and MCF-7 cells were treated for 3 h with 120 µM Pep-1-Phor21 (maximum concentration used in the dose-response analysis, Alamar Blue, Figure 4a). MDA-MB 231 and LM2 cells were treated with Pep-1-Phor21 at 24 µM (maximal effective concentration against LM2, as determined in dose-response analysis, Alamar Blue, Figure 4a). Pep-1-Phor21 had a significant cytotoxic effect only against IL-13Rα2-expressing TNBC cells (MDA-MB-231, LM2; relative cytotoxicity = 85.2% ± 5.4 and 96.9% ± 0.38, respectively, versus non-treated cells). Data = mean value ± SEM of three independent experiments (* *p* ≤ 0.05; ** *p* ≤ 0.01; *** *p* ≤ 0.001; **** *p* ≤ 0.0001).

Having validated its selectivity, Pep-1-Phor21 activity was further tested against breast cancer cell lines representative of TNBC and non-TNBC phenotypes and of defined IL-13Rα2 expression. When grown as monolayers, IL-13Rα2-negative non-malignant MCF-10A cells or malignant non-TNBC MCF-7 cells did not exhibit any appreciable loss of viability (Alamar Blue assay) after treatment with Pep-1-Phor21 (dose-response range = 0–120 µM) or its constitutive peptide subunits (Pep-1, Phor21) for 24 h (Figure 4a). In contrast, IL-13Rα2-positive TNBC-type cell lines, MDA-MB-231 and LM2, were highly sensitive to Pep-1-Phor21 treatment and their sensitivity increased as the time of incubation with Pep-1-Phor21 was increased. Notably, LM2 cells, which exhibited the most prominent surface IL-13Rα2 expression, were the most susceptible to Pep-1-Phor21 (Figure 4b). Furthermore, the measurement of cell death associated with a loss of cell membrane integrity (CellTox Green assay, detects the dead cell DNA) showed that only Pep-1-Phor21 had a demonstrable cytotoxic effect on IL-13Rα2-positive cell lines (Figure 4c) with LM2 cells again being the most susceptible (24 µM Pep-1-Phor21, 3 h: relative cytotoxicity = 96.9% ± 0.4 of maximal activity). In contrast, Pep-1-Phor21 (≤120 µM) did not exhibit appreciable cytotoxic activity against IL-13Rα2-negative cells (MCF-10Aand MCF-7). Since the 3 h treatment seems to be optimal for analyzing the dose-dependent effect of the peptide on the tumor cell lines used in the study, we used the treatment of peptides for 3 h in further experiments unless otherwise indicated.

**Figure 5:**
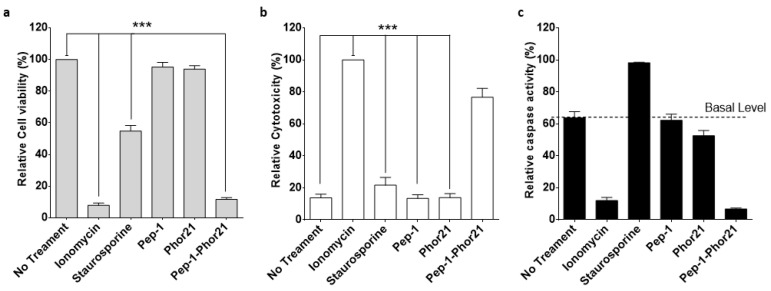
Characterization of the cell-killing mechanism utilized by Pep-1-Phor21. MDA-MB-231 cells were treated with peptides: Pep-1-Phor21, Pep-1, or Phor21 (20 µM); staurosporine (10 µM); or ionomycin (150 µM). After 6 h, cell viability (**a**), cytotoxicity (**b**), and apoptosis (**c**) were assessed by ApoTox-Glo^TM^ Triplex Assay. Individual Pep-1 or Phor21 peptides were inactive against MDA-MB-231 cells. In contrast, all other treatments induced a rapid loss of cell viability (**a**). Pep-1-Phor21 or ionomycin induced a significant increase in relative cytotoxicity, whereas staurosporine did not have a significant cytotoxic effect (Pep-1-Phor21: 76.7% ± 5.6; staurosporine: 21.7% ± 4.88, relative to non-treated cells: 13.8% ± 2.3) (**b**). In contrast, only staurosporine induced an increase in caspase 3/7 activity (34.6% ± 4.9 detectable increase above baseline), consistent with the induction of apoptosis in target cells (**c**). Data = mean value ± SEM of three independent experiments (*** *p* ≤ 0.001).

To more precisely delineate the mode of cell killing, we concomitantly measured the cell viability, cytotoxicity, and apoptosis (ApoTox-Glo Triplex Assay) of IL-13Rα2-positive TNBC MDA-MB-231 cells after treating them with Pep-1-Phor21 (20 µM). As comparative controls, cells were treated with the cytotoxic compound ionomycin (150 µM), or the apoptosis inducer staurosporine (10 µM). The exposure of MDA-MB-231 cells to either Pep-1-Phor21 or control compounds produced a significant loss of viability (loss of internal live-cell protease activity, Figure 5a). However, only Pep-1-Phor21 or ionomycin (but not staurosporine) induced a significant increase in cytotoxicity (increased dead-cell protease activity, released from cells with impaired membrane integrity), consistent with primary necrosis (Figure 5b). In contrast, only staurosporine preferentially activated cellular apoptosis, as demonstrated by increased caspase 3/7 activation above baseline activity (Figure 5c). The Pep-1-Phor21 or ionomycin-treated cells showed lower caspase activity than untreated or Pep-1- or Pho21-treated cells, which is probably due to the loss of cellular components by necrosis. Therefore, Pep-1-Phor21 may be particularly effective against TNBC-type tumors that express high levels of IL-13Rα2. Furthermore, Pep-1-Phor21 has a cytolytic mode of action, rapidly inducing necrotic cell death.

### 3.4. Epigenetic Modulation of IL-13Rα2 Expression in Breast Cancer Cells

**Figure 6:**
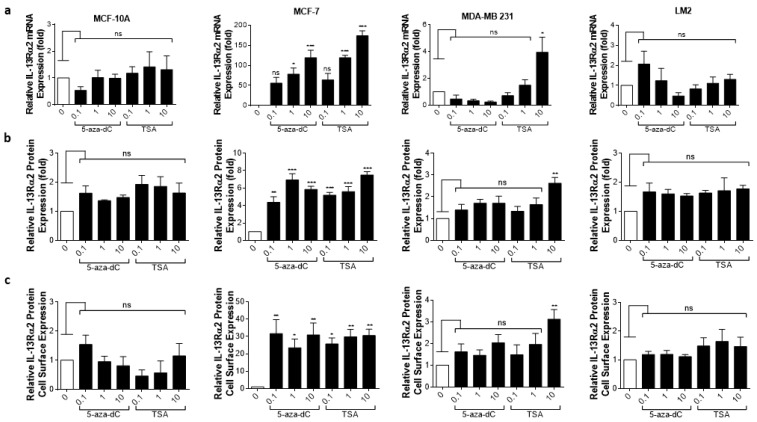
Epigenetic modulation of IL-13Rα2 expression in breast cancer cells. Non-malignant breast epithelial (MCF-10A), non-TNBC (MCF-7), and TNBC (MDA-MB 231, LM2) cell lines were treated with an HDAC inhibitor (TSA) or with a DNMT inhibitor (5-aza-dC) for 24 h and IL-13Rα2 expression at the mRNA, total protein, and cell-surface levels were subsequently measured by RT-PCR (**a**), Western blot (**b**), and cell-based ELISA (**c**), respectively. Treatment with either epigenetically active compound induced detectable levels (all assays) of IL-13Rα2 expression in MCF-7 cells, whereas expression was not altered in MCF-10A cells (remaining IL-13Rα2-negative). In IL-13Rα2-positive cells, 10 µM TSA significantly upregulated IL-13Rα2 expression in MDA-MB-231 cells, as measured in all assays. Data = mean value ± SEM of three independent experiments (* *p* ≤ 0.05; ** *p* ≤ 0.01; *** *p* ≤ 0.001; ns = non-significant; relative to untreated cells).

Given previous reports that IL-13Rα2 expression can be upregulated in certain cancers via epigenetic mechanisms [19], we sought to clarify whether IL-13Rα2 expression could be similarly enhanced in breast cancer cells, sensitizing them to Pep-1-Phor21 in the process. In particular, we determined the effects of treatment with an HDAC inhibitor (TSA), or with a DNMT (5-aza-dC) on IL-13Rα2 expression. The treatment of IL-13Rα2-negative non-TNBC MCF-7 cells with either TSA or 5-aza-dC for 24 h induced the detectable expression of IL-13Rα2 mRNA (Figure 6a), total IL-13Rα2 protein (Figure 6b), and cell-surface IL-13Rα2 expression (Figure 6c) in a dose-dependent manner. In contrast, this induction was not demonstrable in non-malignant MCF-10A cells. Although the TNBC-type cell lines already expressed relatively high levels of IL-13Rα2, significantly increased expression was detectable in 10µM TSA-treated MDA-MB-231 cells, with the LM2 sub-line exhibiting further marginal increases in expression.

**Figure 7:**
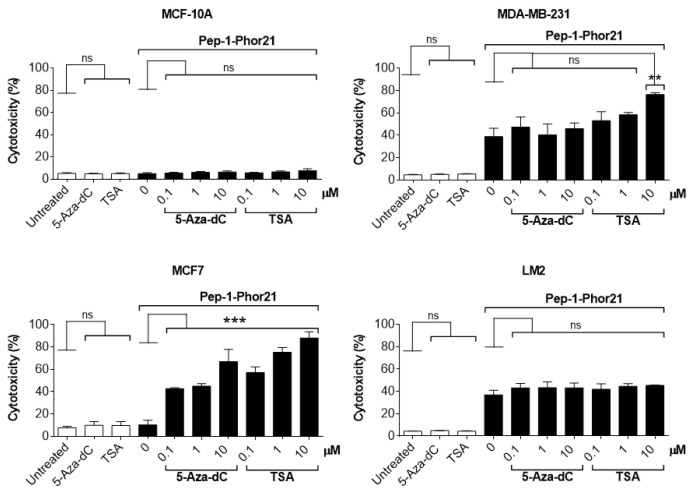
Effect of HDAC or DNMT inhibitors on Pep-1-Phor21-induced cytotoxicity. All cell lines were treated with 0–10 µM TSA or 5-aza-dC for 24 h. MCF-10A, MCF-7, MDA-MB-231, and LM2 cells were subsequently treated with 50 µM, 20 µM, 10 µM, or 0.5 µM Pep-1-Phor21, respectively, for 3 h, and their cytotoxicity was determined (CellTox assay). Pep-1-Phor21 had a demonstrable cytotoxic effect on non-TNBC MCF-7 cells only after pre-treatment with TSA- or 5-aza-dC. Similarly, Pep-1-Phor21 exhibited a significant increase in cytotoxic activity against TNBC MDA-MB-231 cells when pre-treated with 10 µM TSA. Data = mean value ± SEM from three independent experiments (** *p* ≤ 0.01; *** *p* ≤ 0.001; ns = non-significant; relative to untreated cells).

Given that HDAC or DNMT inhibition selectively enhanced IL-13Rα2 expression in tumor cells, we further determined whether this effect translated into increased sensitivity to Pep-1-Phor21. Exposure to the epigenetic modulators themselves (up to 10 µM TSA or 5-aza-dC for 24 h) did not produce any cytotoxic effect. However, consistent with their effects on IL-13Rα2 expression, TSA or 5-aza-dC pre-treatments induced non-TNBC MCF-7 cells to become sensitive to Pep-1-Phor21 (1.0µM 5-aza-dC or 1.0µM TSA, followed by 20µM Pep-1-Phor21: relative cytotoxicity = 84.7% ± 4.5, and 81.9% ± 4.3, respectively; Figure 7). IL-13Rα2-negative MCF-10A cells remained resistant to Pep-1-Phor21 after pre-treatment with either TSA or 5-aza-dC. Consistent with IL-13Rα2 expression data, only 10 µM TSA was able to significantly increase the sensitivity of MDA-MB-231 cells to Pep-1-Phor21. Therefore, TSA or 5-aza-dC can selectively upregulate IL-13Rα2 expression in breast cancer cells (particularly in non-TNBC), increasing their subsequent susceptibility to targeting via Pep-1-Phor21.

### 3.5. Targeting Breast Cancer Spheroids with Pep-1-Phor21

**Figure 8:**
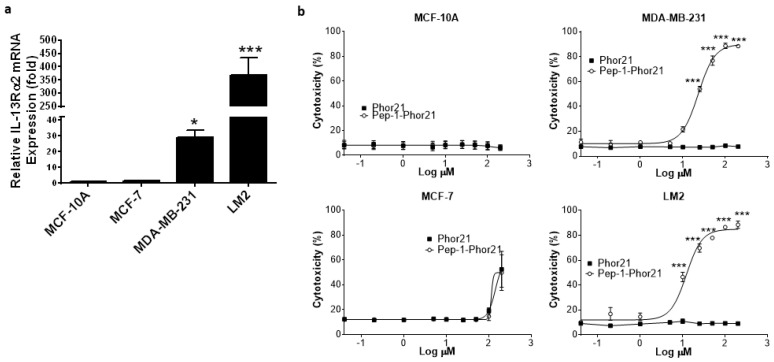
IL-13Rα2 expression in breast cancer spheroids and targeting with Pep-1-Phor21. (**a**) IL-13Rα2 mRNA expression was determined by RT-PCR in the indicated cell lines cultured as 3-D spheroids for 48 h (fold-change relative to MCF-10A cells). (**b**) Established spheroids (48 h) were treated for 3 h with the indicated peptides (dose-response range = 0–120 µM) and their cytotoxicity was assessed (CellTox assay). Only IL-13Rα2-positive, TNBC spheroids exhibited susceptibility to the cytotoxic effect of Pep-1-Phor21 (MDA-MB-231, LM2; IC_50_ = 22.98 µM ± 1.5, 12.22 µM ± 2.5, respectively). Data = mean value ± SEM of three independent experiments (* *p* ≤ 0.05; *** *p* ≤ 0.001).

To more rigorously test the activity of Pep-1-Phor21, we utilized breast cancer cells cultured as spheroids, which exhibit growth characteristics more consistent with solid tumors [43]. All cell lines cultured in the spheroid format maintained their relative IL-13Rα2 expression status (Figure 8a) and, consistent with this, Pep-1-Phor21 had a dose-dependent cytotoxic effect only on MDA-MB-231 or LM2 spheroids (Figure 8b). However, calculated IC_50_ values for Pep-1-Phor21 activity against spheroids were moderately higher than those observed under monolayer conditions—MDA-MB-231, IC_50_ = 22.98 µM ± 1.5; LM2, IC_50_ = 12.22 µM ± 2.5—consistent with the moderately lower IL-13Rα2 mRNA expression levels measured in spheroid cultures (Figure S1). As observed with monolayer cells, the Phor21 lytic peptide subunit displayed no significant cytotoxic activity against spheroid cultures.

**Figure 9:**
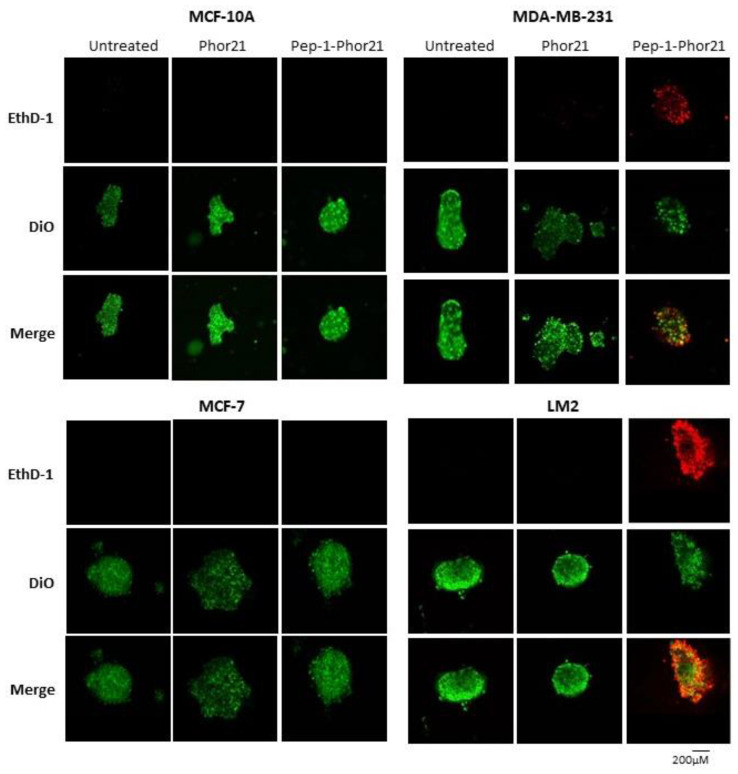
Confocal cell imaging of breast cancer spheroids treated with Pep-1-Phor21. Indicated cell lines established as spheroids (48 h) were treated with Pep-1-Phor21 or Phor21 (3 h, 30 µM) and assessed for the presence of live and dead cells using confocal fluorescent microscopy. IL-13Rα2-positive TNBC spheroids (MDA-MB-231, LM2) exhibited diffuse dead cell staining (red fluorescence, EthD-1) with the concomitant disruption of spheroid integrity after Pep-1-Phor21 treatment. Some foci of live cells (green fluorescence, Vybrant DiO) were detectable within the core of the disrupted spheroid after 3 h. In contrast, IL-13Rα2-negative MCF-10A breast epithelial and non-TNBC MCF-7 cells exhibited only viable cell staining with no observable loss of spheroid structure post-treatment.

**Figure 10:**
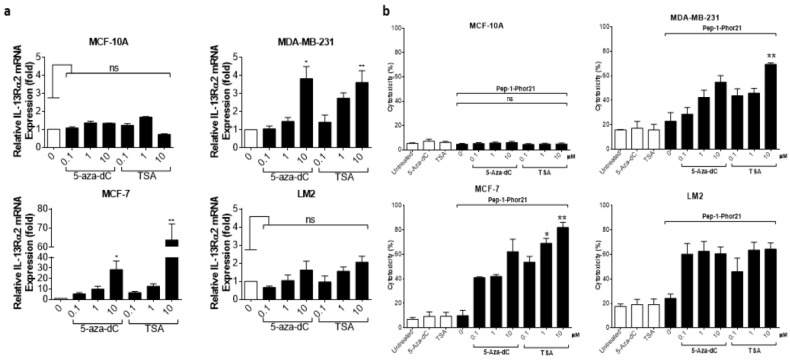
Epigenetic modulation of IL-13Rα2 expression in spheroids and targeting with Pep-1-Phor21. (**a**) Established spheroids (48 h) were treated with either 5-aza-dC or TSA (−10 µM, 24 h), and relative IL-13Rα2 mRNA expression was determined by RT-PCR (fold-change relative to expression in untreated spheroid cells). Whilst non-malignant MCF-10A spheroids remained refractory to treatment, both compounds significantly upregulated IL-13Rα2 expression in non-TNBC MCF-7 spheroids. Similarly, 10µM 5-aza-dC/TSA significantly upregulated IL-13Rα2 expression in TNBC MDA-MB-231 spheroids. (**b**) Spheroids pre-treated with 5-aza-dC or TSA (dose-response range 0–10 µM) were subsequently treated with Pep-1-Phor21 (MCF-10A, MCF-7, MDA-MB-231, and LM2 were treated with 100 µM, 50 µM, 25 µM, and 10 µM, respectively, based on pre-determined IC_50_ values for each spheroid type, Figure 8b) for 3 h, and relative cytotoxicity was evaluated (CellTox assay, relative to untreated spheroids). Data = mean value ± SEM of three experiments (* *p* ≤ 0.05; ** *p* ≤ 0.01; ns = not significant).

To visualize cell killing, confocal fluorescence microscopy was performed on breast cancer spheroids treated with Pep-1-Phor21. This analysis revealed that only IL-13Rα2-positive (TNBC) spheroids exhibited significant dead-cell staining (EthD-1) with the concomitant disruption of spheroid integrity after Pep-1-Phor21 treatment (Figure 9). After 3 h of treatment, dead-cell staining was present throughout the disrupted spheroid with only a few remaining live cells (Vybrant DiO staining) detectable within the inner core of the spheroid. Furthermore, given the potentially altered growth characteristics of cancer cells in spheroids, we further tested the ability of HDAC or DNMT inhibitors to modulate IL-13Rα2 expression. Indeed, as with monolayer cells, TSA or 5-aza-dC treatments significantly increased IL-13Rα2 mRNA expression in non-TNBC MCF-7 spheroids, whereas MCF-10A spheroids maintained their resistance to the modulating effects of these compounds (Figure 10a). Only treatment with 10µM TSA or 5-aza-dC significantly increased IL-13Rα2 expression in TNBC MDA-MB-231 spheroids. Notably, this up-regulation of IL-13Rα2 expression concomitantly enhanced the sensitivity of spheroids to subsequent Pep-1-Phor21 treatment in that non-TNBC MCF-7 spheroids developed sensitivity to Pep-1-Phor21-mediated cytotoxicity (Figure 10b). MDA-MB-231 (TSA- or 5-aza-dC-treated) and LM2 (TSA-treated) spheroids also exhibited a further significant increase in relative sensitivity to Pep-1-Phor21. Therefore, IL-13Rα2 expression in breast cancer spheroids is selectively amenable to epigenetic upregulation and Pep-1-Phor21 rapidly disrupts spheroid integrity, efficiently killing multiple layers of IL-13Rα2-positive tumor cells.

## 4. Discussion

Although the 5-year survival rate for breast cancer has significantly improved, in part due to the development of endocrine-based therapies [44], significant challenges to successful therapy remain, in that breast tumors exhibit heterogeneous phenotypes, including receptor-positive (ER^+^, PR^+^, HER2^+^), triple-negative (ER^−^, PR^−^, HER2^−^), and combinations of these variants. Therefore, selecting appropriate treatments can be problematic, and to compound this, the development of drug resistance poses a major hurdle to achieving a permanent cure [45]. TNBC poses particularly significant challenges in that these aggressive tumors are refractory to hormone receptor-targeting strategies, with treatment predominantly confined to a combination of surgery, radiotherapy, and chemotherapy with cytotoxic drugs. Improved therapies for TNBC-type tumors are therefore required [46], and we hypothesized that our cytolytic peptide-based approach to targeting IL-13Rα2-expressing tumors would provide a selective and potentially more effective strategy.

IL-13Rα2 is reportedly over-expressed in a variety of malignancies [47], and targeting this receptor, therefore, has garnered considerable interest as a possible therapeutic strategy [16,24]. With regard to TNBC, previous studies have shown an association between predominant IL-13Rα2 expression and metastasis [7,48] and our findings corroborate these findings in that elevated expression of IL-13Rα2 was readily detectable in TNBC-type tumors and cell lines. Furthermore, IL-13Rα2-mediated signaling has been shown to promote the metastasis of ER-negative breast cancer cells [8], and targeting IL-13Rα2 may be a particularly relevant strategy for these metastasis-prone tumors. In formulating an IL-13Rα2-targeting strategy, the Pep-1 sequence motif was selected, as it exhibits a high affinity for IL-13Rα2, binding the receptor at an extracellular site that is distinct from the IL-13-binding region [24] and theoretically avoiding off-target effects. Moreover, incorporating the cell-surface-active lytic Phor21 motif predicted that in contrast to exotoxin-drug conjugates, internalization would not be required for the cell-killing activity of Pep-1-Phor21. Indeed, we demonstrate that Pep-1-Phor21 rapidly mediates cytolytic-based necrosis, consistent with previous studies in which lytic peptides of this type caused rapid membrane disruption [27,49]. Given these properties, Pep-1-Phor21 should, therefore, circumvent many intracellularly active drug-resistance mechanisms. We have shown recently that Pep-1-Phor21 kills pancreatic cancer cells by targeting IL-13Rα2 since the cells lost sensitivity to this hybrid peptide when the receptor expression was downregulated in these cells by using its siRNA [31]. While this work was in progress, two more anti-cancer peptides that target IL-13Rα2 were identified [50,51]. The first peptide, which is derived from the D1 domain of IL-13Rα2, has been shown to exhibit therapeutic activity against metastatic colorectal cancer by competitively inhibiting IL-13 binding to IL-13Rα2 and thereby the receptor signaling [50]. The second peptide, which was isolated as an IL-13Rα2 binding peptide by screening a T7 phage display library, has shown effects in glioblastoma therapy when conjugated with a lytic peptide [51].

As a relatively small peptide (34 amino acids, molecular weight = 3.5 kDa), Pep-1-Phor-21 should be able to penetrate vascular endothelium and solid tumor structures. However, standard monolayer cultures poorly represent the physical barriers encountered within the in vivo tumor microenvironment and, consequently, can be poor predictors of drug efficacy [52]. To address such limitations, multi-cellular spheroid models have been developed [53,54,55,56], which, in addition to presenting more relevant tumor cell gene expression patterns [54], also permit the analysis of the presence of extracellular matrix proteins and cell–cell interactions [57]. The latter is particularly relevant in that tight junctions between different cell types within tumors can limit efficient drug penetration [58]. This study reports data from such a 3-D spheroid model which utilized live cell imaging in conjunction with cytotoxicity assays to quantitatively analyze spheroid growth. Pep-1-Phor21-mediated cytotoxicity was less efficient against spheroids (moderately higher IC_50_ values), and although the requirement for the penetrance of multiple cell layers could be partly responsible for this observation, IL-13Rα2 mRNA expression was also moderately reduced in spheroid-cultured cells (Figure S1). Indeed, given the rapid physical disruption of spheroid integrity, we speculate that the latter observation forms the predominant basis for the differences in cytotoxic effect observed between monolayer and spheroid cultures. This reduction in IL-13Rα2 expression is potentially explained by the findings of previous studies in that the central layers of multi-cellular spheroids were shown to be hypoxic [59,60] and, notably, IL-13Rα2 mRNA expression in glioblastoma cells was reportedly decreased under hypoxic conditions [61]. Whether hypoxia within the breast cancer spheroids accounts for the moderate reduction in IL-13Rα2 expression requires further investigation. Nevertheless, despite such obstacles, diffuse dead cell staining and spheroid rupture were rapidly detectable post-treatment, indicating that Pep-1-Phor21 could efficiently penetrate breast cancer spheroids, selectively killing IL-13Rα2-expressing tumor cells in the process.

Epigenetic changes, including DNA methylation and histone modifications, have been linked to the progression of breast cancer with markedly different epigenetic profiles discernible between the early and late stages of breast cancer [21,22,23]. Of note, it has been shown that ER expression can be re-established in breast cancer cells through treatment with HDAC inhibitors [23], and we demonstrate in this study that IL-13Rα2 expression is similarly regulated in that the inhibition of either histone deacetylation or DNA methylation produced an upregulation of IL-13Rα2 expression, enhancing consequent susceptibility to Pep-1-Phor21 treatment. Encouragingly, this modulation of IL-13Rα2 expression was only demonstrable in malignant cells, pointing to potentially specific tumor cell targeting when using Pep-1-Phor21 in combination with epigenetically active anti-cancer compounds. The reason for this selective effect of epigenetic modifiers on breast cancer cells remains unclear. However, studies on other cancers revealed that IL-13Rα2 expression requires AP-1/c-jun signaling and this pathway was shown to be quiescent in non-malignant cells, which, similar to the findings of this study, were refractory to HDAC inhibitor-induced IL-13Rα2 expression [19]. Whether these specific signaling events also account for the variable expression of IL-13Rα2 in the various sub-types of breast cancer requires further study. It is also possible that combinations of epigenetically active drugs will more potently upregulate IL-13Rα2 in breast cancer cells and, in this regard, it is notable that combination therapy with 5-azacytidine and the HDAC inhibitor Entinostat is currently undergoing evaluation in clinical trials as a therapy for advanced breast cancer [62].

## 5. Conclusions

We have shown that IL-13Rα2 exhibits potential as a therapeutic target, particularly for TNBC types of breast cancer. Furthermore, IL-13Rα2-expressing cells are efficiently targeted with Pep1-Phor21, even within solid spheroid cultures, consistent with the small molecular weight of this type of drug and its rapid cytolytic mode of action. Importantly, drug-induced cytolysis could be enhanced by treatment with epigenetically active anti-cancer compounds, suggesting that the combination adjuvant therapy of Pep1-Phor21 with such compounds may be a more effective treatment for non-TNBC-type tumors.

## Data Availability

The data can be shared upon request.

## Supplementary Materials

The following supporting information can be downloaded at: https://www.mdpi.com/2072-6694/15/10/2772/s1, Figure S1: RT-PCR analysis of IL-13Ra2 mRNA expression in non-malignant (MCF-10A), non-TNBC (MCF-7), and TNBC (MDA-MB-231) cells cultured either as monolayers (2-D) or as 3-D spheroids. RNA was extracted from cells after 3 days of culture and subjected to RT-PCR using β-actin expression as a housekeeping control. Data = mean value ± SEM of three independent experiments (* *p* ≤ 0.05), fold change relative to expression in MCF-10A cells cultured as monolayers; Figure S2: Time-dependent analysis of breast non-tumor (MCF-10) and cancer (MCF-7, and MDA-MB-231) cell growth using Alamar Blue assay. MCF-10A, MCF-7, and MDA-MB-231 were seeded into a 96-well µClear^®^ half area black plate at cell concentrations of 10,000, 20,000, 40,000, and 80,000 cells per well. After 24 h, the medium was replaced with a medium containing Alamar Blue. Fluorescence was measured using 544 nm (excitation) and 590 nm (emission) settings on a POLARstar Omega microplate reader (BMG LABTECH). Measurements were taken on days 0, 1, 2, 3, and 4. File S1: original-images.
