# Correction: Jannoo et al. Targeting and Sensitization of Breast Cancer Cells to Killing with a Novel Interleukin-13 Receptor α2-Specific Hybrid Cytolytic Peptide. *Cancers* 2023, *15*, 2772

**DOI:** 10.3390/cancers16051006

**Published:** 2024-02-29

**Authors:** Riaz Jannoo, William Walker, Venkateswarlu Kanamarlapudi

**Affiliations:** 1 UCL ECMC GCLP Facility, UCL Cancer Institute, University College London, London WC1E 6DD, UK; m.jannoo@ucl.ac.uk; 2 Institute of Life Science, School of Medicine, Swansea University, Singleton Park, Swansea SA2 8PP, UK; w.walker@swansea.ac.uk

In the original publication [1], there was a mistake in Figures 4 and 9 and reference [43] when published. The headers for the top two tables were inaccurate in Figure 4b. Incorrect images were used for MCF-10A (untreated), MCF-7 (Phor21) and MDA-MB-231 (Phor21) in Figure 9. The corrected Figure 4 and Figure 9 and Reference [43] appears below. 

Reference [43]: Gkretsi, V.; Stylianou, A.; Louca, M.; Stylianopoulos T. Identification of Ras suppressor-1 (RSU-1) as a potential breast cancer metastasis biomarker using a three-dimensional in vitro approach. *Oncotarget* **2017**, *8*, 27364–27379. https://doi.org/10.18632/oncotarget.16062.

The authors apologize for any inconvenience caused and state that the scientific conclusions are unaffected. This correction was approved by the Academic Editor. The original publication has also been updated.

## Figures and Tables

**Figure 4:**
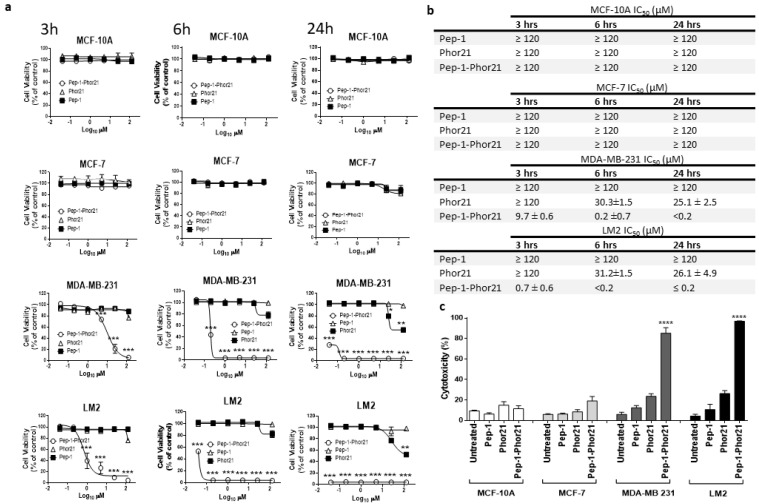
Treatment of representative breast cancer cell lines with Pep-1-Phor21. (**a**) Dose-dependent effect of Pep-1-Phor21 (O), Pep-1 (■), or Phor21 (∆) on the viability of non-tumorigenic (MCF-10A), non-TNBC (MCF-7), and TNBC cells (MDA-MB 231, LM2). Cells were treated with different concentrations of Pep-1-Phor21 (effective concentration range = 0–120 µM, a 5-fold serial dilution) and their viability was assessed after 3 h, 6 h, or 24 h of the treatment by Alamar Blue assay. (**b**) The IC_50_ of peptides for various cell lines for different incubation times. (**c**) The cytotoxic effect of individual peptides on cell lines was also assessed by CellTox assay. MCF-10A and MCF-7 cells were treated for 3 h with 120 µM Pep-1-Phor21 (maximum concentration used in the dose-response analysis, Alamar Blue, Figure 4a). MDA-MB 231 and LM2 cells were treated with Pep-1-Phor21 at 24 µM (maximal effective concentration against LM2, as determined in dose-response analysis, Alamar Blue, Figure 4a). Pep-1-Phor21 had a significant cytotoxic effect only against IL-13Rα2-expressing TNBC cells (MDA-MB-231, LM2; relative cytotoxicity = 85.2% ± 5.4 and 96.9% ± 0.38, respectively, versus non-treated cells). Data = mean value ± SEM of three independent experiments (* *p* ≤ 0.05; ** *p* ≤ 0.01; *** *p* ≤ 0.001; **** *p* ≤ 0.0001).

**Figure 9:**
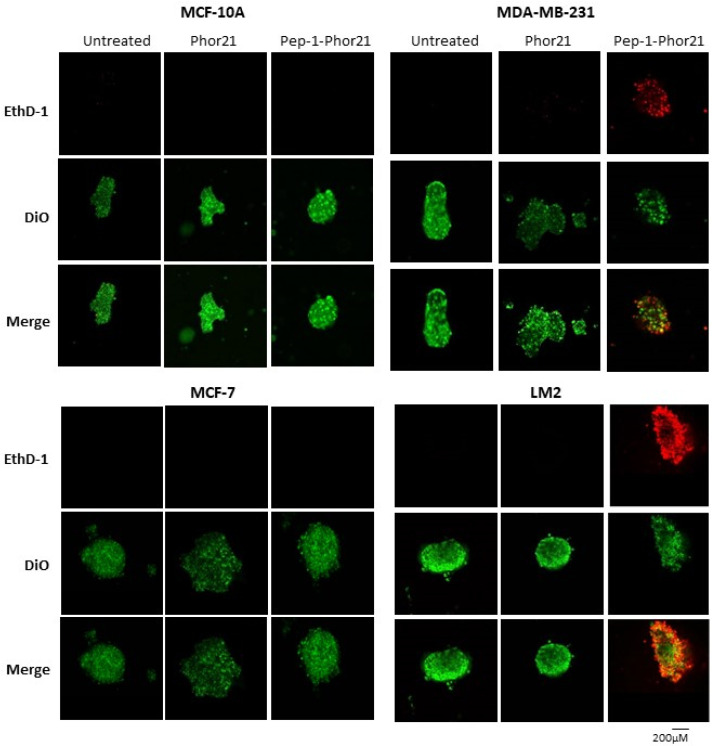
Confocal cell imaging of breast cancer spheroids treated with Pep-1-Phor21. Indicated cell lines established as spheroids (48 h) were treated with Pep-1-Phor21 or Phor21 (3 h, 30 µM) and assessed for the presence of live and dead cells using confocal fluorescent microscopy. IL-13Rα2-positive TNBC spheroids (MDA-MB-231, LM2) exhibited diffuse dead cell staining (red fluorescence, EthD-1) with the concomitant disruption of spheroid integrity after Pep-1-Phor21 treatment. Some foci of live cells (green fluorescence, Vybrant DiO) were detectable within the core of the disrupted spheroid after 3 h. In contrast, IL-13Rα2-negative MCF-10A breast epithelial and non-TNBC MCF-7 cells exhibited only viable cell staining with no observable loss of spheroid structure post-treatment.
