# Supplementary figures and images for: Targeting and Sensitization of Breast Cancer Cells to Killing with a Novel Interleukin-13 Receptor α2-Specific Hybrid Cytolytic Peptide
Source: Cancers (Basel). 2023 May 16;15(10):2772. doi: 10.3390/cancers15102772 (PMC10216279; doi:10.3390/cancers15102772)

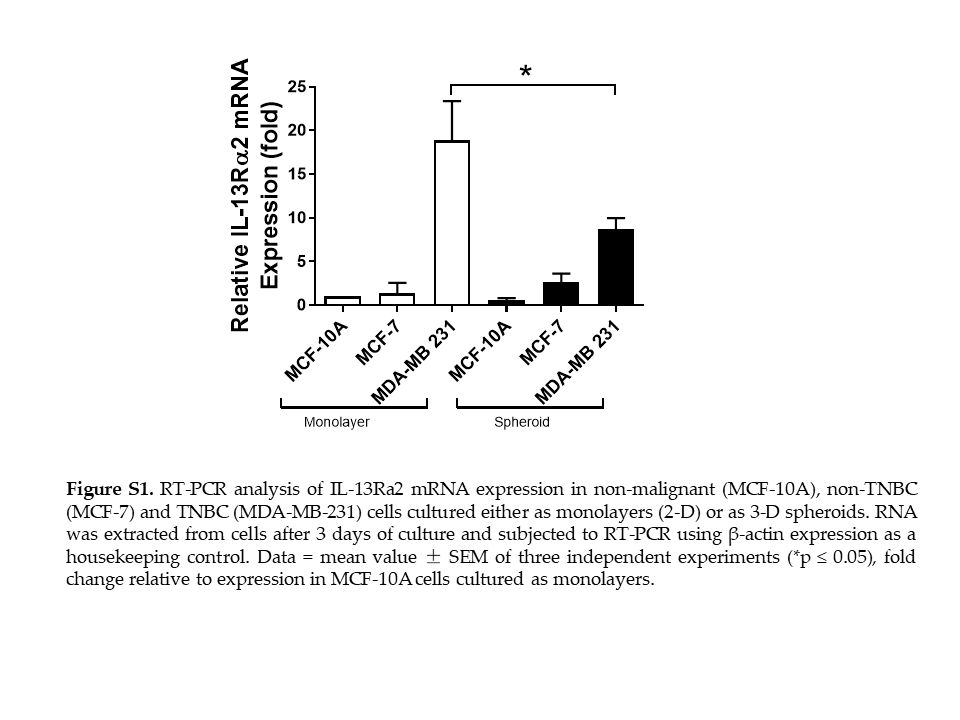

Supplement: Supplementary file 1 [file cancers-15-02772-s001.zip › Figure S1.tif]

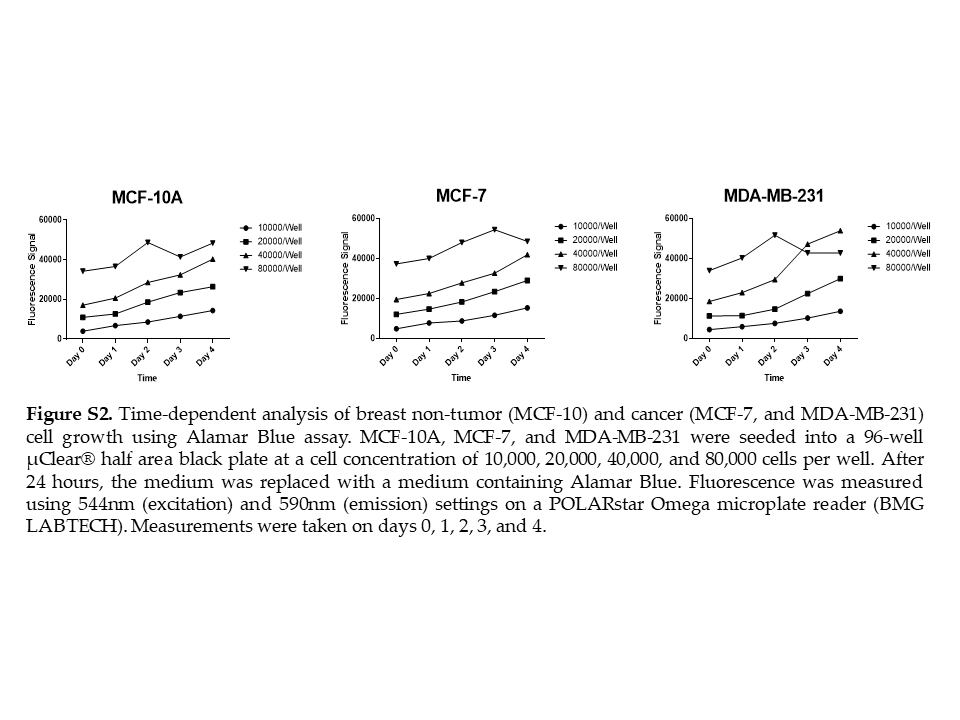

Supplement: Supplementary file 1 [file cancers-15-02772-s001.zip › Figure S2.tif]

Figure 2b

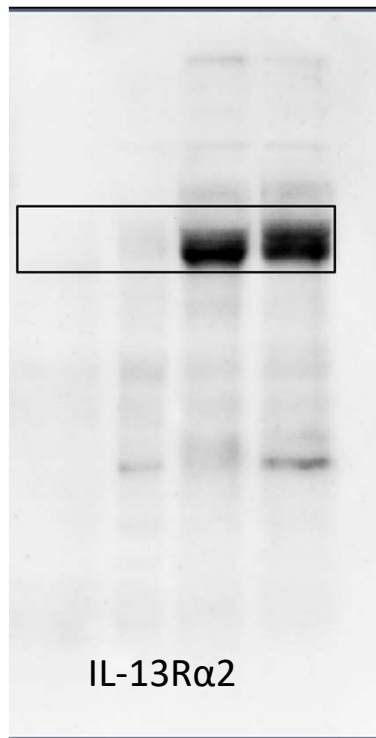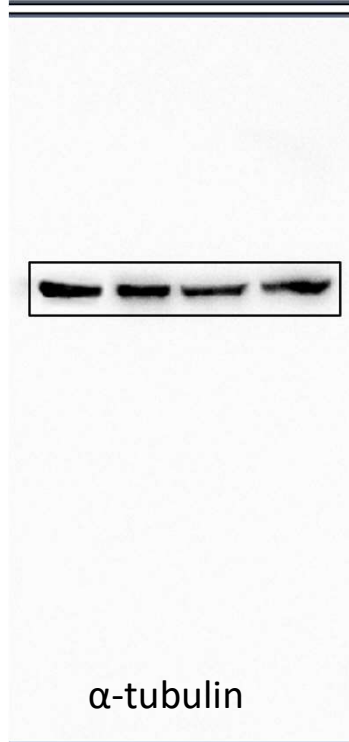

Figure 3a

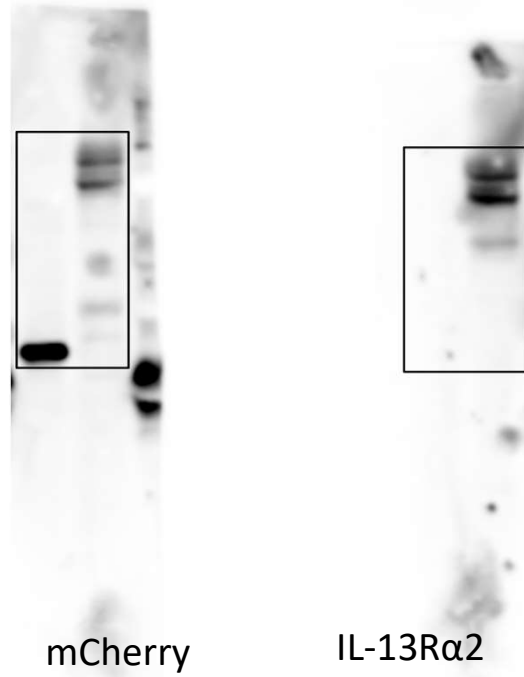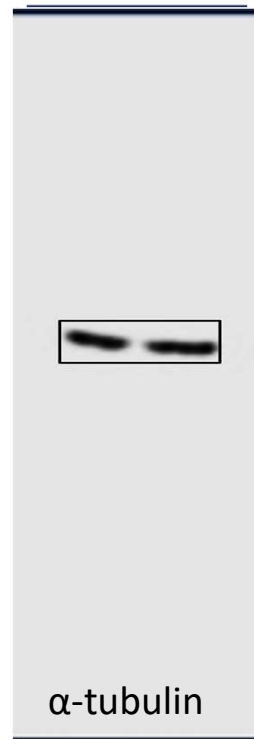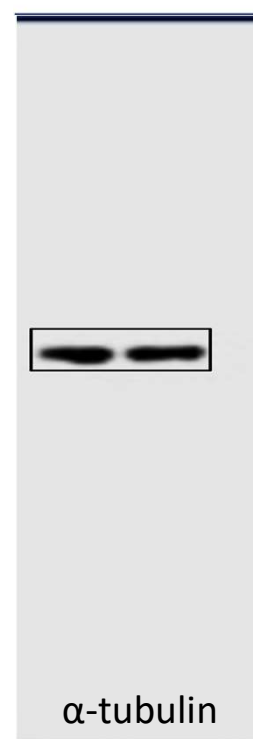

Supplement: Supplementary file 1 [file cancers-15-02772-s001.zip › file S1-original-images.pdf]
